# Continent‐wide population genomic structure and phylogeography of North America’s most destructive conifer defoliator, the spruce budworm (*Choristoneura fumiferana*)

**DOI:** 10.1002/ece3.5950

**Published:** 2020-01-07

**Authors:** Lisa M. Lumley, Esther Pouliot, Jérôme Laroche, Brian Boyle, Bryan M. T. Brunet, Roger C. Levesque, Felix A. H. Sperling, Michel Cusson

**Affiliations:** ^1^ Royal Alberta Museum Edmonton AB Canada; ^2^ Laurentian Forestry Centre Natural Resources Canada Quebec City QC Canada; ^3^ Université Laval Quebec City QC Canada; ^4^ University of Alberta Edmonton AB Canada

**Keywords:** *Choristoneura*, comparative phylogeography, genotyping‐by‐sequencing, *Picea glauca*

## Abstract

The spruce budworm, *Choristoneura fumiferana*, is presumed to be panmictic across vast regions of North America. We examined the extent of panmixia by genotyping 3,650 single nucleotide polymorphism (SNP) loci in 1975 individuals from 128 collections across the continent. We found three spatially structured subpopulations: Western (Alaska, Yukon), Central (southeastern Yukon to the Manitoba–Ontario border), and Eastern (Manitoba–Ontario border to the Atlantic). Additionally, the most diagnostic genetic differentiation between the Central and Eastern subpopulations was chromosomally restricted to a single block of SNPs that may constitute an island of differentiation within the species. Geographic differentiation in the spruce budworm parallels that of its principal larval host, white spruce (*Picea glauca*), providing evidence that spruce budworm and spruce trees survived in the Beringian refugium through the Last Glacial Maximum and that at least two isolated spruce budworm populations diverged with spruce/fir south of the ice sheets. Gene flow in the spruce budworm may also be affected by mountains in western North America, habitat isolation in West Virginia, regional adaptations, factors related to dispersal, and proximity of other species in the spruce budworm species complex. The central and eastern geographic regions contain individuals that assign to Eastern and Central subpopulations, respectively, indicating that these barriers are not complete. Our discovery of previously undetected geographic and genomic structure in the spruce budworm suggests that further population modelling of this ecologically important insect should consider regional differentiation, potentially co‐adapted blocks of genes, and gene flow between subpopulations.

## Graphical abstract

We examined the population genomic structure of the spruce budworm, *Choristoneura fumiferana*, and found three spatially structured subpopulations: Western (Alaska, Yukon), Central (southeastern Yukon to the Manitoba–Ontario border) and Eastern (Manitoba–Ontario border to the Atlantic). Geographic differentiation in the spruce budworm parallels that of its principal larval host, white spruce (*Picea glauca*), providing evidence that spruce budworm and spruce trees survived in the Beringian refugium through the Last Glacial Maximum and that at least two isolated spruce budworm populations diverged with spruce/fir south of the ice sheets. Gene flow in the spruce budworm may also be affected by mountains in western North America, habitat isolation in West Virginia, regional adaptations, factors related to dispersal, and proximity of other species in the spruce budworm complex.

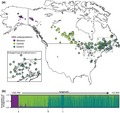

## 1 INTRODUCTION

Large, spatially continuous populations have played a fundamental role in developing evolutionary theory, shaping our understanding of biogeography, population genetics, and speciation (Darwin, 1872; Fisher, 1930; MacArthur & Wilson, 1967; Mayr, 1963; Wright, 1931). Populations with continental‐scale geographical distributions, large population sizes, and high mobility theoretically undergo high gene flow indicative of panmictic, clinal, or weakly differentiated populations (Mayr, 1963, but see Barton & Charlesworth, 1984 and references therein). Yet, if such populations are cyclical and undergo endemic‐to‐epidemic changes in size, they may be subject to founder effects during endemic periods, allowing genetic differentiation (Carson, 1968; Templeton, 1980, but see Barton & Charlesworth, 1984). If they are herbivorous, there is also a possibility that they have an intimate connection to their host plant, which may indeed lead to genetic diversification and adaptive radiation in association with adaptive divergence of the host plant to variable habitats (Gloss, Groen, & Whiteman, 2016). Continental‐scale, cyclical, herbivorous insect pests may provide an appropriate natural model to help understand or verify these underlying mechanisms. In addition, from a pest management perspective, studying continental‐scale population genomics of an insect defoliator, to delimit spatially defined subpopulations, can enable an assessment of regional parameters and biological traits for each subpopulation (e.g., host plant association, population size, response to climatic factors, response to habitat composition and connectivity, and dispersal mechanisms) to create regionally refined life‐history summaries, management protocols, and predictions of population outbreak dynamics. These climatic and biological parameters also affect gene flow, exchange of advantageous alleles, and/or adaptive divergence (e.g., Garant, Forde, & Hendry, 2007; Levins, 1964; Piálek & Barton, 1997; Rāsānen & Hendry, 2008; Sexton, Hangartner, & Hoffmann, 2014). For pest species, this may include the exchange of heritable traits contributing to large‐scale damage in outbreaks, or advantageous alleles in warming environments (Visser, 2008). Population genomics also allows us to study the genomic architecture underlying differences in subpopulations, such as co‐adapted blocks of genes, which may have large consequences on the rate of local adaptation, provide potential targets for genetic‐based pest management, and contribute to modeling and monitoring.

**Figure 1:**
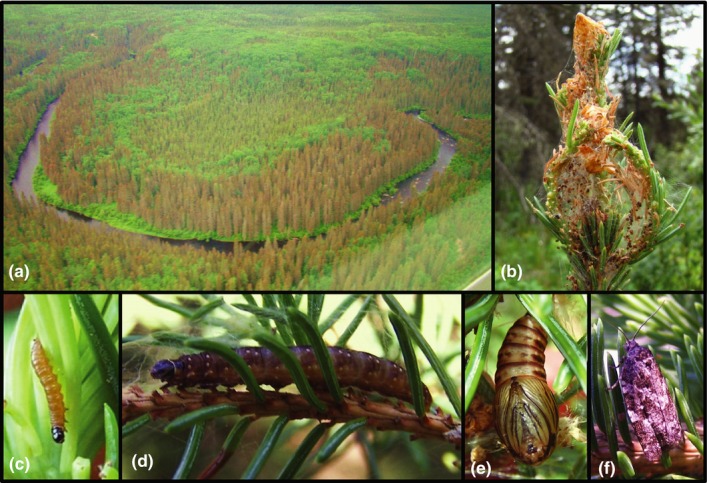
Photographs of *Choristoneura fumiferana* and its damage. (a) Aerial photograph of a large‐scale spruce budworm outbreak. (b) Damage caused by larval feeding. (c) Third instar larva. (d) Sixth instar larva. (e) Pupa. (f) Adult. Photographs: LM Lumley

The spruce budworm, *Choristoneura fumiferana* (Clemens, 1865) (Figure 1), has had a long history of research, including the identification of genetically inherited, environmentally associated traits that have an impact on the propensity for, and scale of, outbreaks. Harvey (1983a) reported that the number and size of eggs laid by females vary across the geographic range of spruce budworm, with fewer, larger, and heavier eggs in the northwest and more, smaller, and lighter eggs in the southeast. Harvey demonstrated that egg weight is heritable (Harvey, 1983b, 1985) and further showed a positive relationship between mean egg weight and overwintering survival of second‐instar larvae in prolonged cold storage that emulated the longer winters of higher latitudes (Harvey, 1985). With climate change and warming environments, a southeast to northwest movement of genes related to egg weight and egg numbers could increase the propensity for outbreak in the northwest, by allowing females to lay more eggs with increased overwintering survival of the resulting larvae in warmer, shorter winter conditions. Locating barriers to gene flow may therefore allow forest managers to evaluate the likelihood of movement of these and other adaptive genes across the landscape, and better assess the need for mitigation.

Spruce budworm interactions with its larval host plants (spruces and firs) involve a cyclical 30–40 year outbreak pattern, with the high‐density outbreak period typically lasting 10 years or longer. An outbreak in the 1970s and 1980s affected over 55 million hectares of forest in eastern Canada (Blais, 1983), while a new outbreak initiated in Quebec in 2006 has so far defoliated over 8.1 million hectares in this province (Ministère des Forêts de la Faune et des Parcs, 2018) and is now spreading to New Brunswick. The spruce budworm is economically devastating to *Picea glauca* (Moench) Voss (white spruce) and *Abies balsamea* (L.) Mill. (balsam fir). It also frequently feeds on *Picea rubens* Sarg. (red spruce) and *P. mariana* (Mill.) Britton, Sterns and Poggenburg (black spruce), and is occasionally found on *P. engelmannii* Parry ex Engelm. (Engelmann spruce), *Abies lasiocarpa* (Hooker) Nuttall (subalpine fir), *Larix laricina* (Du Roi) K. Koch (Eastern larch) and *Pinus* species (Volney, 1989). White spruce has evolved some resistance to spruce budworm herbivory (Lamara et al., 2018; Mageroy et al., 2015), and this is reflected in the population genetic structure of the tree (Méndez‐Espinoza et al., 2018; Parent et al., 2017). Spruce budworm behavior has changed in the presence of its host plants or some of their components (e.g., waxes and sugars) (Albert, 1991; Daoust et al., 2010; Ennis, Despland, Chen, Forgione, & Bauce, 2017), indicating that it has evolved adaptive, genetically driven traits associated with herbivory.

Several recent studies have focused on the phylogeography and population genetic structure of spruce and fir species (Cinget, Gérardi, Beaulieu, & Bousquet, 2015; de Lafontaine, Turgeon, & Payette, 2010; Jaramillo‐Correa, Beaulieu, Khasa, & Bousquet, 2009 and references therein; Potter, Frampton, Josserand, & Nelson, 2010). Yet, we have limited knowledge of spruce budworm phylogeography and population genetic structure, or how phylogeographic patterns compare with those of its host plants. Recent work on the spruce budworm has mostly focused on species delimitation, mechanisms of speciation, and phylogeography among species within the spruce budworm pest complex (Blackburn et al., 2017; Brunet et al., 2017; Dupuis et al., 2017; Fagua, Condamine, Brunet, et al., 2018; Fagua, Condamine, Dombroskie, et al., 2018; Lumley & Sperling, 2010, 2011a, 2011b), which includes twelve species and subspecies, or fifteen “biotypes” as per Volney and Fleming (2007). Larval‐host interactions appear to be strongly tied to speciation in the spruce budworm species complex (Fagua, Condamine, Brunet, et al., 2018; Fagua, Condamine, Dombroskie, et al., 2018). For *C. fumiferana*, May, Leonard, and Vadas (1977) studied electrophoretic variation of loci in spruce budworm collected in northern Maine and found no significant population differentiation. James et al. (2015) used microsatellite markers to study the population genetics of spruce budworm within the Border Lakes region of Ontario and Minnesota to detect dispersal by comparing larvae (residents) to adults captured in pheromone traps (potential migrants). Recently, Larroque et al. (2019) used SNP markers to detect spatiotemporal variation in population genetic structure during spruce budworm population outbreaks in Quebec. The most comprehensive study to examine *C. fumiferana* population genetic structure across its geographic range is that of Harvey (1996). His work suggested that the species was near panmixia, with a single allozyme locus exhibiting a significant northwest to southeast genetic cline. Here, we re‐examine this hypothesis, capitalizing on next‐generation sequencing methods to determine whether additional genetic markers can detect geographic structure in spruce budworm. Subpopulations would affect gene flow and adaptive potential, thereby providing information that can be incorporated into management strategies.

By studying the population genetic structure of *C. fumiferana* across its geographic range, our goal was to determine (a) whether there are subpopulations, (b) where geographic or genomic barriers to gene flow may limit the potential for movement of adaptive traits, and (c) why barriers, if any, have appeared.

## 2 MATERIALS AND METHODS

### 2.1 Insect collections

Specimens were collected as larvae and reared to the adult stage, or collected as adults in pheromone traps. Most collections were obtained in 2012, when provincial forestry divisions collected adult moths with pheromone traps, or larvae on branches of *Abies balsamea* (L.) Mill. (balsam fir) and *Picea glauca* (Moench) Voss (white spruce), from locations that were approximately 200 km apart, from Alberta to the Atlantic. These were sent to the Laurentian Forestry Centre (LFC), where pheromone‐trap specimens were frozen at −20°C, and branches were searched for *C. fumiferana* larvae that were subsequently reared to adults in individual plastic cups with foliage from their respective host plant. Additional wild‐caught samples (pheromone trapped or reared) collected between 2007 and 2014 were also included to increase our sampling range to Alaska, Newfoundland, and the United States, as well as to fill in geographical gaps (Table S1). For each sample locality, 25 individuals were randomly chosen for molecular analysis where possible. If fewer than 25 individuals were collected, we included as many samples as were available.

### 2.2 DNA extraction, library preparation, and sequencing

DNA extractions were completed using DNeasy 96 Blood & Tissue Kits (Qiagen) with RNaseA (Qiagen) digestion. Seven specimens collected in Alberta were extracted following the protocols of Brunet et al. (2017). For the remaining specimens, for each individual the wings (pheromone‐trap specimens) or wings and abdomen (reared specimens) were clipped off and the remaining tissue was placed in a collection microtube containing a 5‐mm stainless steel bead. The tissue was ground into a fine powder by two rounds of the microtubes being placed in liquid nitrogen for 30 s and then disrupted using a TissueLyser II bead mill (Qiagen) for 15 s at 23–24 Hz. The microtubes were centrifuged briefly to remove tissue from the tube lids before the lids were removed to add buffer ATL and proteinase K, to reduce possible cross‐contamination between samples. DNA was then extracted according to manufacturer's instructions, with the following modifications. When transferring the lysate of each sample to the well of the DNeasy plate (step 9), only the top liquid portion was transferred and the particulate accumulated toward the bottom of the well was avoided as it negatively interacted with subsequent steps and resulted in poor‐quality DNA. For the final step, three elutions of 50 µl AE buffer, each incubated for 2–5 min before centrifugation, gave a final eluted volume of 150 µl. DNA purity was assessed using a NanoDrop 8,000 spectrophotometer (Thermo Fisher Scientific, Waltham, Massachusetts, USA), and only samples with a 260/280 and 260/230 absorbance ratio ≥1.7 were processed further. DNA concentration for all samples was quantified with a Fluoroskan Ascent Labsystem (Thermo Electron Corporation, Vantaa, Finland) using the Quant‐iT™ PicoGreen® dsDNA Assay Kit (Invitrogen) and then normalized to 10 ng/µl per individual. Genotyping‐by‐sequencing (GBS), a restriction digest protocol, was completed through preparation of *PstI*‐*MspI* libraries (96‐plex; Poland, Brown, Sorrells, & Jannink, 2012) at the Plateforme d’Analyses Génomiques de l’Université Laval (Quebec City, Canada), with a duplex‐specific nuclease treatment (Zhulidov et al., 2004) and a one‐nucleotide (C) complexity reduction PCR (Sonah et al., 2013) to increase depth of coverage. Single‐end, 100‐bp read sequencing of these libraries was performed using an Illumina HiSeq2000 at the McGill University—Génome Québec Innovation Centre (Montreal, Canada).

### 2.3 Processing of Illumina sequences for SNP discovery

Raw GBS data were processed with the Fast‐GBS pipeline (Torkamaneh, Laroche, Bastien, Abed, & Belzile, 2017) using the spruce budworm genome (Larroque et al., 2020: spruce budworm bw6 genome assembly) for reference alignment. This included demultiplexing, concatenating samples that were sequenced twice, trimming adapter sequences, and variant‐calling. Twenty‐nine samples were removed because they had too few reads, leaving 2089 samples for alignment. After initial alignment, the pipeline initiates Platypus (Rimmer et al., 2014) to improve the quality of mapping and to call all variants. The pre‐imputation output from Platypus was filtered using *VCFtools* (Danecek et al., 2011) to: (a) remove indels (‐‐*remove‐indels*) and variants that were not biallelic SNPs (*‐‐min‐alleles 2 ‐‐max‐alleles 2*), exclude loci that did not pass all Platypus filters (*‐‐remove‐filtered‐all*), exclude loci with read depth of <3 reads (*‐‐minDP 3*), minor allele count <3 (*‐‐mac 3*), minimum quality score <30 (*‐‐minQ 30*), and/or if genotyped in <50 percent of individuals (*‐‐max‐missing 0.5*), and exclude genotypes with quality score of <30 (*‐‐minGQ 30*); (b) remove individuals missing >70% data and then remove loci with minor allele frequency (maf) <0.005; (c) remove individuals with >65% missing data and then remove loci with maf <0.005 and genotyped in <90% individuals; (d) remove individuals with >30% missing data and then remove loci with maf <0.005 and genotyped in <95% individuals; (e) remove individuals with >20% missing data and then remove loci genotyped in <96% of individuals; and (f) remove individuals with >15% missing data. The sequential removal of individuals and loci described in steps (b) to (f) allowed us to maintain more markers and individuals, as it reduced the likelihood that high‐quality SNPs were removed due to poor‐quality individuals and vice versa. The subsequent dataset, comprised of 1975 individuals with ≤15% missing data and 3,676 SNPs with <4% missing data, was analyzed using the R package *adegenet* (Jombart, 2008) to calculate observed heterozygosity for each allele across samples. We removed 26 loci with heterozygosity >0.5 as they may be paralogs (Hohenlohe, Amish, Catchen, Allendorf, & Luikart, 2011). We used *adegenet* to test for Hardy–Weinberg equilibrium (HWE) and did not remove any additional loci as none were out of HWE (*p* < .01) at more than 60% of collection locations.

### 2.4 Spruce budworm population analyses

We ran several analyses to study how individuals clustered without any a priori information for geography or sampling locality. We analyzed individual admixture coefficients with the *snmf* function from the LEA package (Frichot & François, 2015) in R, as well as in *structure v2.3.4.* (Pritchard, Stephens, & Donnelly, 2000). Both *snmf* and *structure* are useful in estimating and visualizing population genetic structure, with *snmf* using sparse nonnegative matrix factorization algorithms and *structure* using Bayesian algorithms. For *snmf*, we set the function to analyze K = 1–15 for 10 iterations each, then interpreted the cross‐entropy plot, calculated best K, and visualized the best run plotted as a bar chart. For *structure*, we used the admixture model, with 10,000 burn‐in and 10,000 MCMC after burn‐in for 5 iterations each of K = 1–10. We used *Structure Harvester* (Earl & vonHoldt, 2012) to calculate *ΔK* (Evanno, Regnaut, & Goudet, 2005) and *Clumpak* (Kopelman, Mayzel, Jakobsson, Rosenberg, & Mayrose, 2015) to visualize the K1‐10 barplots. We consulted the inferred ancestry of individual's output from *structure* for K = 3, as K = 3 was calculated as the most likely number of subpopulations and assigned each individual to a subpopulation based on the most supported inferred cluster. We then created a pie chart map in R to spatially represent the *structure* results for K = 3. *Structure* also calculated the net nucleotide distance among each pair of clusters, expected heterozygosity between individuals in the same cluster, and mean value of *F*_st_ for each cluster. These values help to visualize the levels of genetic difference among and within clusters.

Using the R package *adegenet* (Jombart, 2008), we used *find.clusters* (max.n.clust = 128, number PCs retained = 1975 and 500) to determine the most likely number of subpopulations (K). We used principal component analysis (PCA) to visualize genetic clustering of individuals, with no scaling, and missing values replaced by the mean allele frequency. In addition, we used *adegenet* to calculate *F*_st_ values between pairs of localities using Nei's estimator of pairwise *F*_st_. Here, we removed collection locations with <18 individuals to reduce the possibility of spurious results due to these localities containing a low number of individuals. This dataset included 59 collection locations from across the spruce budworm range, with a total of 1551 individuals and 3,650 polymorphic SNPs.

### 2.5 Linkage block detection

The subclustering pattern of individuals within the central and eastern geographic regions along the PCA axes 1 and 2 was indicative of the data containing SNPs within a linkage block (see also Trevoy, Janes, Muirhead, & Sperling, 2019). To investigate this further, we reran PC analysis with specimens from the western geographic region (Alaska, Yukon) removed, to obtain SNP loading values associated with the three subclusters along axis 1. We then sorted the SNP loading values from highest to lowest to view the top contributing SNPs, graphed the loading values, and determined whether there was a set of high contributing SNPs both similar to each other in loading values and comparatively higher than the next consecutive contributing SNP, as documented in Trevoy et al. (2019). We then searched for these SNPs in the published *C. fumiferana* linkage map data (Picq et al., 2018b: supplementary information “File_S2_Picq_et_al_2018_20180626,” worksheet “Linkage map details”) to determine the linkage group and chromosome location for these SNPs. One SNP was not included in the linkage map, and we conducted additional comparisons of this SNP and its flanking sequence with the Picq et al. (2018a) data and a newer genome assembly (October 2018). We used BioEdit v7.2.0 (Hall, 1999) to create a local nucleotide database file of all scaffolds within the October 2018 assembly, and then completed two separate local BLASTn searches, one search for the current SNP sequence and the second search for all sequences documented by Picq et al. (2018a). We then: (a) documented the best October 2018 scaffold match for the current SNP sequence; (b) searched this scaffold within the BLASTn results for the Picq sequences; (c) documented the Picq SNPs associated with this scaffold; and (d) searched for these SNPs and their associated linkage group and chromosome location information in Picq et al. (2018a) supplementary information “File_S2_Picq_et_al_2018_20180626,” worksheet “Linkage map details.” Upon concluding that the three highest contributing SNPs were of the same linkage group, we repeated PCA analyses for all individuals with the three linkage block SNPs removed (1975 individuals, 3,647 SNPs) to determine how the removal of these loci affected population clustering.

Upon determining that the three highest contributing SNPs were contained within the same linkage group, we used the same methods described above to assess the linkage group for the next consecutive highest contributing SNP and continued this assessment of each consecutive SNP until we reached five consecutive SNPs that were not of this same linkage group. We carried out BLASTx searches on the NCBI website (https://blast.ncbi.nlm.nih.gov/Blast.cgi) against the database of nonredundant protein sequences to find matches between the translated sequences flanking these SNPs and published gene annotations. In addition, we assessed the combination of the three linkage block SNPs for each individual within the entire dataset (1975 individuals) to determine whether the SNPs underwent Mendelian inheritance as a unit (i.e., “A/A, A/A, A/A,” “A/B, A/B, A/B,” “B/B, B/B, B/B”) and summarized these data for the western, central, eastern (excluding West Virginia), and West Virginia geographical regions to assess how the pattern of inheritance changes across the geographically structured *C. fumiferana* subpopulations. Geographically associated allele combinations would give evidence that this block of SNPs constitutes an island of differentiation within the species.

## 3 RESULTS

### 3.1 Collections and SNP filtering

In total, 128 collections from 112 separate locations were included in the analyses, sampled across the geographic range of *C. fumiferana,* including 88 adult and 40 larval collections (Table S1). After quality filtering, the resulting SNP dataset was comprised of 1975 individuals genotyped for 3,650 SNP loci, with maximum missing data of 15% for individuals and 4% for loci. *Adegenet* analysis found a total of 1.15% missing data.

### 3.2 Spruce budworm population analyses

**Figure 2:**
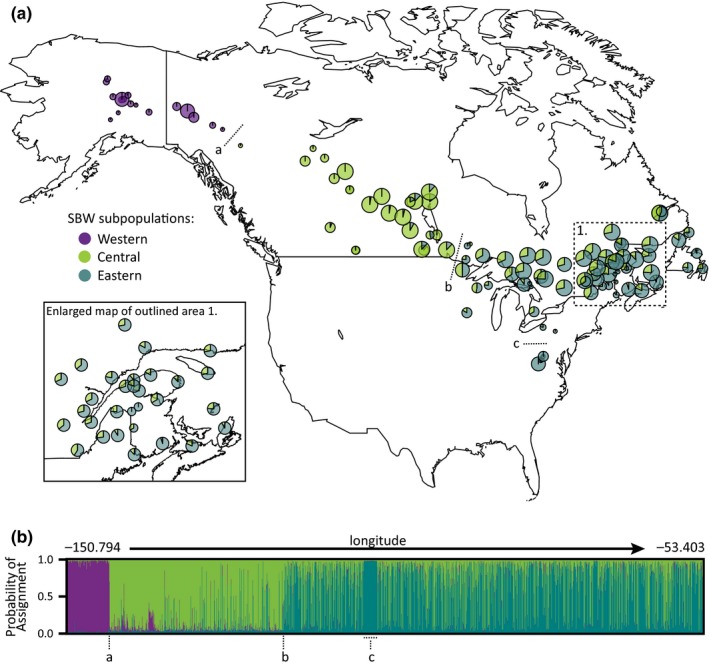
(a) Map of spruce budworm study localities, with each locality represented as a pie chart to indicate number of individuals per locality assigned to each population (K = 3) with *structure* analysis. Pie chart size variation represents number of samples, from smallest circles representing <5 individuals to largest circles representing >25 individuals. The dotted lines labelled by a letter represent barriers to gene flow: a. in southeastern Yukon between Watson Lake and Ross River, b. along the Manitoba–Ontario border, and c. between West Virginia and more northern localities within the eastern region. (b) Bar plot from *structure* analysis (K = 3) representing probability of SNP assignment for 1975 spruce budworm individuals. Individuals are sorted by longitude, with far left representing most western samples (Fish Creek, Alaska) to far right representing most eastern samples (Seal Cove Pond, Newfoundland). Letters along the bottom of the plot represent potential geographic barriers to gene flow: a. between Watson Lake and Ross River, Yukon; b. along the Manitoba–Ontario border; and c. the dotted line delimiting all individuals sampled from West Virginia

Both *structure* and *snmf* calculated K = 3 as the most likely number of subpopulations. For *structure*, *ΔK* was calculated from the likelihood results which indicated that the most likely number of subpopulations was three (Figure S1; mean likelihood = −1898060.98, *ΔK* = 21.830705). We used the inferred ancestry of individuals from the K = 3 *structure* results to assign each individual to a subpopulation based on the most supported inferred cluster. We created a pie chart map (Figure 2a) to spatially represent the individual assignments to these three subpopulations, which we refer to as “Western” (Alaska and Yukon), “Central” (mostly southeastern Yukon to Manitoba), and “Eastern” (mostly east of Manitoba, from Ontario and Minnesota to the Atlantic).^1^ Percent assignment for each individual as represented by the *structure* bar chart (Figure 2b, Figure S2) indicates that the Western subpopulation is the most distinct and has the highest genetic integrity. A few individuals located within the western geographic region contain loci with genotypes that would be assigned to the Central or Eastern subpopulations, but well below 50%, meaning that all individuals still assigned to the Western subpopulation. In comparison, 6.9% of individuals located in the central geographic region assigned to the Eastern subpopulation, and 25.7% of individuals located in the eastern geographic region assigned to the Central subpopulation. No individuals within the central and eastern regions assigned to the Western subpopulation. The *structure* analyses reported an allele frequency divergence among subpopulations (net nucleotide distance) of 0.0015 (Western and Central), 0.0021 (Western and Eastern), and 0.0004 (Central and Eastern). Average distances (expected heterozygosity) between individuals within each subpopulation were 0.0709 (Western), 0.0691 (Central), and 0.0678 (Eastern). The mean value of *F*_st_ within each subpopulation was 0.0381 (Western), 0.0011 (Central), and 0.0041 (Eastern).

**Figure 3:**
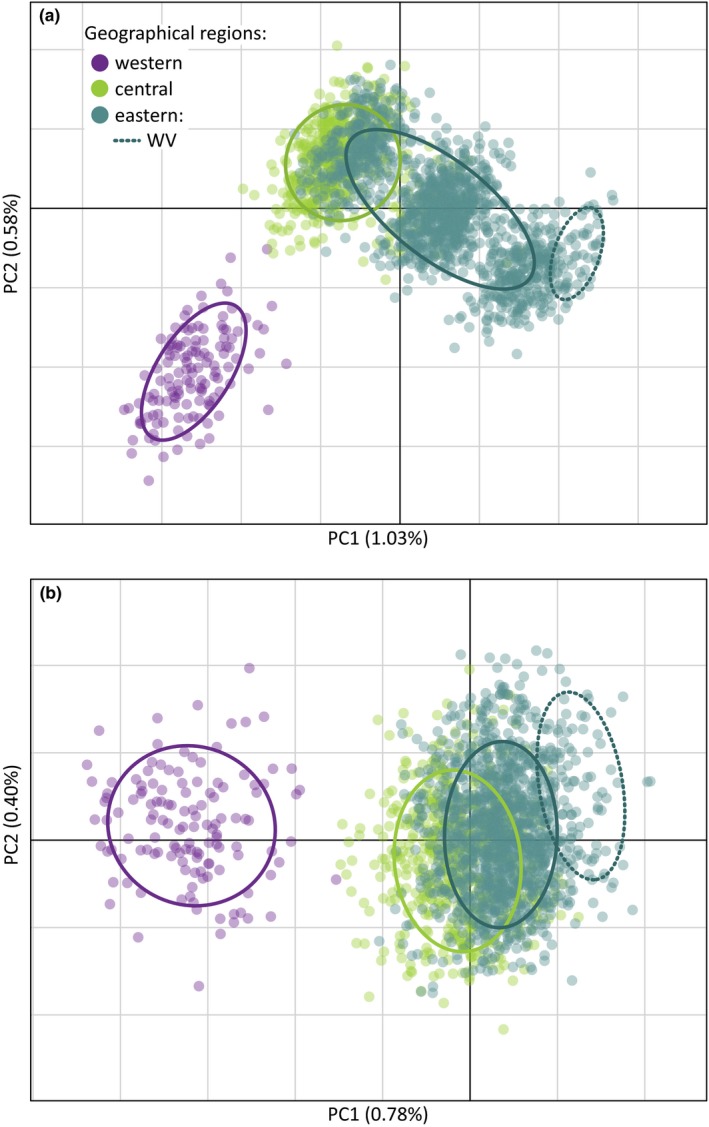
Principal component analysis (PCA) of 1975 spruce budworm individuals genotyped for (a) 3,650 SNPs, and (b) 3,647 SNPs (three highest contributing loci removed). Each point is an individual, colored according to geographical region in which it was collected. A 95% inertia ellipse is drawn for each region, with the ellipse for West Virginia (WV) shown separately for the eastern region. The provinces or states included in each region are: western—Alaska, Yukon; central—Alberta, Manitoba, Saskatchewan; eastern—Iowa, Maine, Michigan, Minnesota, New Brunswick, Newfoundland and Labrador, Nova Scotia, Ontario, Pennsylvania, Quebec, Wisconsin, West Virginia

Using *adegenet*, the *find.clusters* function indicated that the most likely number of subpopulations is three. Principal component analysis (PCA) revealed two main genetic clusters (Figure 3a), one cluster representing all specimens from Alaska and Yukon except for one individual collected near Watson Lake, YT, and the second cluster representing specimens from all remaining sample localities. The second cluster appeared to have three overlapping subclusters that represent a northwest to southeast geographical cline. These three subclusters mostly include sample localities from: (a) YT (Watson Lake only), Alberta, Saskatchewan, Manitoba; (b) Ontario, Quebec, Nova Scotia, New Brunswick, Newfoundland, Labrador, Minnesota, Iowa, Wisconsin, Michigan, Pennsylvania, Maine; and (c) West Virginia. Nonetheless, there was overlap, with some individuals collected in Alberta, Saskatchewan, and Manitoba placed within the middle subcluster and some individuals collected further east (except West Virginia) placed within the first subcluster. Additional results associated with this pattern are documented in the *Linkage block detection* section below.

Population pairwise *F*_st_ values calculated for collection localities with >18 individuals revealed similar results. The highest differentiation (0.0402–0.0439) was found between populations in Alaska and the Yukon (Western) and West Virginia (Eastern). Overall, pairwise population differentiation ranged from 0.0178 to 0.0283 (mean = 0.0223) between the Western and Central subpopulations, from 0.0197 to 0.0439 (mean = 0.0268) between the Western and Eastern subpopulations, and from 0.0068 to 0.0285 (mean = 0.0129) between the Central and Eastern subpopulations. Pairwise population differentiation for locations collected within the same subpopulation ranged from 0.0126 to 0.0175 (mean = 0.0152) for the Western subpopulation, from 0.0060 to 0.0147 (mean = 0.0097) for the Central subpopulation and from 0.0061 to 0.0260 (mean = 0.0122) for the Eastern subpopulation.

### 3.3 Linkage block detection

The pattern of three subclusters along the PCA axes 1 and 2, for individuals from the central and eastern geographic regions, suggests that the data contain SNPs within a linkage block that changes in frequency from Central to East. When we removed all individuals from the Western region and reran PCA, the three highest contributing loci were similar in axis 1 loading values (range: 0.33245–0.32102) and displayed comparatively higher values than the fourth locus (0.19763) (Figure S3). Upon searching for the three linkage block SNPs within the Picq et al. (2018a) data, two SNPs (scf1481820_32081 and scf1429067_18653) belonged to linkage group 4 (LG4), while the third SNP (Path5088_60893) did not have a match on the linkage map. However, through additional comparisons with a newer genome assembly (October 2018), we found that the latter SNP and three other LG4 SNPs in the Picq dataset (Path4531_84585, Path4531_84655, Path4531_84546) colocalized on scaffold 425 of the newer assembly, indicating that SNP Path5088_60893 was also on LG4. The Picq dataset indicates that the three SNPs are all on separate scaffolds located on LG4 at 40.68 cM.

When we removed the three linkage block SNPs from the PC analysis, the three subclusters that appeared with the full complement of 3,650 SNPs for the Central and Eastern subpopulations were reduced to a single overlapping cluster (Figure 3b). Upon assessment of SNPs with consecutive highest PC loading values, we found that nine of the top ten SNPs were located on seven scaffolds contained on LG4 (Table S2). BLASTx searches for the three linkage block SNPs gave no significant hits for SNP scf1481820_32081 and SNP Path5088_60893. SNP scf1429067_18653, however, significantly matched glycerol‐3‐phosphate dehydrogenase [NAD(+)], which has been shown to play roles in insect diapause (Hayakawa & Chino, 1982) and flight (Colgan, 1992). BLAST searches gave significant hits for five of the remaining six top contributing SNPs on LG4 (Table S2), with significant matches to: glycerol‐3‐phosphate dehydrogenase [NAD(+)], ultraviolet‐sensitive opsin, and zinc finger SWIM domain‐containing protein 8. Ultraviolet‐sensitive opsin is associated with vision, forming photopigments sensitive to ultraviolet wavelengths (Zuker, Montell, Jones, Laverty, & Rubin, 1987). Zinc fingers bind DNA, RNA, other proteins, or lipids, and are reported to be involved in regulation of gene expression (Klug, 1999).

Upon viewing the alleles for the three linkage block SNPs (in order: Path5088_60893, scf1429067_18653, scf1481820_32081) for all individuals within the dataset, 1933 of the 1975 individuals (98%) contained the combinations A/A, A/A, G/G (all SNPs homozygous: “A‐A‐G”), G/G, C/C, A/A (all SNP homozygous: “G‐C‐A”) or A/G, A/C, and A/G (all SNPs heterozygous: “R‐M‐R”). In the western and central regions, 80.3% and 89.8% of individuals contained the AAG allele combination, respectively (Table S3). In the eastern region, all three allele combinations were common (AAG: 26.4%, RMR: 49.6%, GCA: 22.9%) except in West Virginia where 100% of individuals contained the GCA allele (Table S3). Overall, these results suggest that these three SNPs form part of a linkage block and undergo Mendelian inheritance as a unit.

## 4 DISCUSSION

Delimitation of subpopulations across the geographic range of a pest species identifies the barriers that reduce gene flow between subpopulations and allows a refined understanding of the advantageous alleles, behavior, and life‐history traits that affect the species’ propensity to outbreak. Here, our analyses revealed three main *C. fumiferana* subpopulations: Western (Alaska, Yukon), Central (southeast Yukon to Manitoba), and Eastern (Ontario to Atlantic). Overall, at least three types of isolating barriers—physical, genetic/adaptive, and bioclimatic—may explain the presence of these subpopulations, which we discuss below.

Phylogeographic scenarios hypothesized for white spruce, the most widespread primary larval host of spruce budworm, can likely be extended to *C. fumiferana* because (a) its current geographic range coincides with that of white spruce, (b) it depends on its host plant for survival, and (c) the timing of boreal forest expansion appears to coincide with a time‐calibrated mitogenome phylogeny for the spruce budworm species complex (Fagua, Condamine, Brunet, et al., 2018). The phylogeography of balsam fir can also be considered, but may be less conclusive as this host does not extend the full range of spruce budworm. In turn, our findings for spruce budworm may also be helpful in understanding the phylogeography of its host plants.

The geographic distribution of the three *C. fumiferana* subpopulations closely matches the phylogeographic pattern of white spruce as established by de Lafontaine et al. (2010). These authors hypothesized that white spruce subpopulations diverged during the Last Glacial Maximum (LGM), when ice sheets covered northern North America with land‐based ice at its maximum from at least 22,000 to 19,000 yrs before present (BP) (Yokoyama, Lambeck, De Deckker, Johnston, & Fifield, 2000). *Picea* macrofossils have been found in the Yukon, dated between about 26,000 and 24,500 ^14^C yr BP, but evidence for tree survival through the LGM is still limited (Zazula, Telka, Harington, Schweger, & Mathewes, 2006). The genetically distinct Western subpopulation of spruce budworm provides evidence that both spruce budworm and white spruce forests survived in the Beringian refugium, a region of unglaciated terrain covering parts of Yukon, Alaska, and eastern Siberia, through the LGM (Anderson, Hu, Nelson, Petit, & Paige, 2006; de Lafontaine et al., 2010).

**Figure 4:**
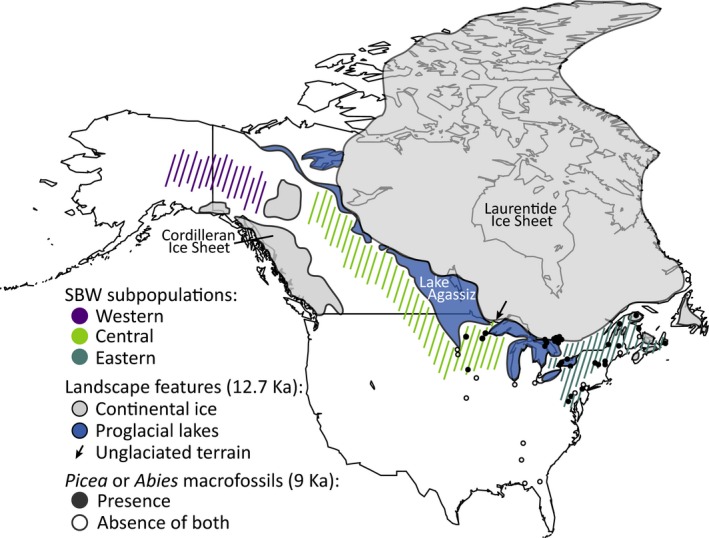
Historical factors that may have contributed to the development and maintenance of three *Choristoneura fumiferana* subpopulations include the Wisconsin continental ice sheets and the large proglacial lakes that formed along the southern border of the Laurentide ice sheet as it melted. Shown here are the proposed continental ice and proglacial lakes between 12.65 and 12.75 Ka (from Murton, Bateman, Dallimore, Teller, & Yang, 2010) that would have created barriers to southern and eastern migration of the Western subpopulation, eastern migration of the Central subpopulation, and western migration of the Eastern subpopulation. These barriers also likely contributed to the population structure of spruce budworm larval host plants; shown are presence (black circles) of *Picea* and/or *Abies* macrofossils at 9 Ka (from Jackson et al., 1997) with absence of both host plants marked by open circles. Hatched, colored regions illustrate the likely occupied and/or available habitat for expansion by spruce budworm subpopulations and their hosts. The black arrow points to the narrow path of terrain between Lake Agassiz and the proglacial Great Lakes that may have allowed for some eastern migration of the Central subpopulation and its larval hosts

The genetic boundary for both spruce budworm and white spruce along, or east of, the Manitoba–Ontario border is not self‐evident, since this region is now connected by contiguous boreal forest. de Lafontaine et al. (2010) proposed that Central and Eastern white spruce subpopulations diverged genetically during the LGM when they were physically isolated in Mississippian and Appalachian glacial refugia, respectively. However, pollen and macrofossil studies of *Picea* conclude that it was widespread and abundant south of the Laurentide ice sheet at the LGM, and physical isolation of *Picea* in separate Mississippian and Appalachian glacial refugia is not apparent in these studies at this time period (Davis, 1983; Jackson, Overpeck, Webb, Keattch, & Anderson, 1997; Ritchie & MacDonald, 1986; Watts, 1983). Therefore, we propose an alternative hypothesis according to which both *Picea glauca* and *C. fumiferana* diverged more recently and further north along their migration routes. Macrofossils dated at 12 and 9 Ka gave evidence for the formation of separate *Picea* populations to the west and east of the proglacial Great Lakes as the glacier retreated northward (Figure 4), and these populations appear to have maintained physical separation until at least 6 to 3 Ka (Jackson et al., 1997). *Abies* also follows this pattern (Jackson et al., 1997), and at least some populations may have a similar, more recent timing of divergence than previously proposed (Cinget et al., 2015). As the continental glacier melted and retreated, glacial Lake Agassiz formed on the uncovered southern regions of Manitoba, western Ontario, Minnesota, and North Dakota. This would have also created a formidable physical barrier to east‐west movement during forest recolonization, with Lake Agassiz covering an estimated 440,000 km^2^ at its greatest extent, for a period that lasted at least 5,000 years (13,000 to 8,200 BP). Lake Agassiz may help explain the presence of more individuals from the Central subpopulation occurring in the eastern region. According to the 12 and 9 Ka maps for *Picea* and *Abies* from Jackson et al. (1997), spruce budworm individuals from the Central subpopulation may have followed their host plants along unglaciated terrain between the eastern side of Lake Agassiz and western side of the proglacial Great Lakes (Figure 4). At this time, the ice sheet and proglacial Great Lakes were still a western barrier to both the trees and the Eastern subpopulation of spruce budworm and, as a consequence, they continued to migrate eastward around the ice sheet toward the Atlantic and northern Quebec (Figure 4).

Current physical barriers to gene flow include mountain ranges between the Western and Central subpopulations. A series of mountain ranges with above‐treeline elevations separates Watson Lake, YT (Central), from the remaining collection localities sampled in the Yukon (Western). Watson Lake is also connected via lower elevation coniferous habitat to the central region. It is possible that there is genetic leakage through to Ross River, YT, which is connected to Watson Lake via the valley between the Selwyn and Pelly Mountains, as the genome of the single individual analyzed from this location contained a higher proportion of inferred ancestry assignment (*structure* analysis) to the Central population than other individuals located in Yukon and Alaska. As our sampling was very limited (*n* = 1 for both Ross River and Watson Lake), these relationships are tentative and further study in this area, northern British Columbia, and Northwest Territories, is needed to provide finer delineation of the Western–Central boundary.

Spruce–fir forests continue to thrive in the higher elevation (above 1,700 m) regions of the Appalachians but have been replaced by deciduous hardwood forests in the surrounding lower elevations. This habitat isolation of West Virginia coniferous forests from forests to the north also appears to have created a physical barrier to spruce budworm gene flow. Individuals studied from the Appalachians of West Virginia have strikingly little admixture (Figure 2; Figure S2) in comparison with individuals collected in other locations of the eastern region and all individuals assigned to the Eastern subpopulation. It is possible that populations in the Appalachians are closest genetically to the historical source of the Eastern subpopulation. The genetic integrity of the West Virginia population suggests that they now have little to no genetic exchange with populations to the north, likely due to the current physical isolation of fir/spruce forest habitat in this region from forests to the north.

Spruce budworm populations can undergo massive outbreaks and have the ability to disperse hundreds of kilometers (Dobesberger, Lim, & Raske, 1983; Greenbank, Schaefer, & Rainey, 1980), and it is thereby unlikely that current subpopulation structure is upheld solely by physical isolation and genetic drift that occurred during the LGM. This applies particularly for the Central and Eastern subpopulations, since (a) a physical boundary is no longer apparent, (b) viable F1 hybrids and backcrossed offspring can be produced by crossing these subpopulations in the laboratory (Picq et al., 2018a), and (c) there are individuals heterozygous for the three linkage block SNPs (“RMR”). In the absence of physical barriers, adaptive barriers may contribute to reduced gene flow. In this scenario, subpopulations reside in different environments and have followed different evolutionary trajectories to adapt and thrive in these local conditions. With no physical barriers, movement of individuals between geographic regions is possible, but the success rate of colonization by hybrids or dispersing individuals may be low either due to reproductive incompatibility (unlikely in this case) or the lack of necessary adaptations to the new environment (Hendry, 2017). There is a striking resemblance between the geographic boundaries of the three spruce budworm subpopulations and the Canadian climate regions (Environment & Climate Change Canada, 2019), with the dividing lines between Western and Central, and Central and Eastern subpopulations corresponding to dividing lines between the Yukon/North B.C. Mountains and Northwestern Forest, and Northwestern Forest and Northeastern Forest, respectively. Researchers have also uncovered a “defoliation belt” (Candau, Fleming, & Wang, 2018), an area that has undergone moderate to severe spruce budworm defoliation at least once between 1941 and 2005. The western edge of this defoliation belt aligns with the border of the Central and Eastern subpopulations and has a lower average climate moisture index (see Candau et al., 2018). Overall, this evidence suggests that spruce budworm subpopulations have likely diverged to adapt to different environmental conditions.

In addition, the discovery of the three SNPs that appear to form a linkage block on linkage group 4 suggests that underlying genetic mechanisms have assisted in maintaining subpopulation boundaries. These behave like a single Mendelian marker with homozygous “AAG” and “GCA” representing what may have been the “pure forms” of the Central and Eastern subpopulations, respectively. A linkage block like this can affect the potential for selection on one trait to pull with it particular variants in other traits. Overall, nine of the top ten contributing SNPs that differentiated the Central and Eastern subpopulations were on linkage group 4, and were located on the chromosome between 37.33 and 40.68 cM. This region of the chromosome may be a genomic outlier region, also coined “island of genomic differentiation” or “speciation island,” containing a barrier locus (or loci) that contributes to reproductive isolation (Trevoy et al., 2019; Turner, Hahn, & Nuzhdin, 2005; Wolf & Ellegren, 2017). It is also possible that this region has arisen through linked selection via genetic drift or background selection, and the associated loci are not necessarily relevant to population divergence (Wolf & Ellegren, 2017). The LG4 SNPs that could be annotated were associated with insect diapause and/or flight (cytoplasmic glycerol‐3‐phosphate dehydrogenase [NAD(+)]), ultraviolet vision (ultraviolet‐sensitive opsin), and DNA‐binding and/or gene expression (zinc finger SWIM domain‐containing protein 8). It is intriguing that genes regulating traits related to overwintering strategies and/or dispersal mechanisms may be linked. Additional work is required to make any further assessment of these observations (see Wolf & Ellegren, 2017).

Along with physical barriers and adaptive divergence, it is likely that bioclimatic factors associated with dispersal may contribute to the geographical distribution of spruce budworm subpopulations, or at least to the movement of their genes. In particular, prevailing westerly winds have likely influenced spruce budworm movement and subsequent gene flow. Our analyses indicate that the Western subpopulation experiences little to no gene flow from the subpopulations to the east, whereas individuals in the province of Alberta, which is the most adjacent region to the Western subpopulation, display a higher proportion of Western‐assigning markers compared to localities further east. This implies the existence of limited eastward gene flow from the Western subpopulation to the Central subpopulation, likely aided by prevailing westerly summer winds. We also observed what could be a west‐to‐east dispersal pattern for the Central and Eastern subpopulations. A lower percentage of individuals within the central (6.9%) versus the eastern (25.7%) geographic regions were assigned to the Eastern and Central subpopulations, respectively. Within these regions, prevailing westerly winds in the summer may aid a west‐to‐east movement in dispersal and increase the likelihood that individuals from the Central subpopulation disperse into the eastern geographic region. This is in agreement with the studies of Anderson and Sturtevant (2011) and Sturtevant et al. (2013) in the Border Lakes region of Ontario and Minnesota, just east of the Manitoba–Ontario border. Using modelling methods, they found adult dispersal patterns to be strongly directional from northwest to southeast throughout an entire dispersal season (Anderson & Sturtevant, 2011). Additional simulations yielded a broader range of dispersal directions, yet still with no dispersal to the west (Sturtevant et al., 2013). Connecting back to the evolution of genetic/adaptive barriers, it is possible that individuals from the Central subpopulation are also not as impeded by bioclimatic conditions in the Eastern region, with Central moths being more likely to colonize successfully upon dispersal compared to individuals from the Eastern subpopulation colonizing westward.

Along with the factors described above, gene flow from other species may influence the genetic diversity of *C. fumiferana*. All species within the spruce budworm species complex can hybridize and produce fertile offspring (Harvey, 1997). Introgression with *C. orae*, *C. occidentalis biennis,* and *C. occidentalis occidentalis* may contribute to the genetic differentiation of *C. fumiferana* along the western limits of its range, and introgression with *C. pinus* may contribute to the genetic differentiation of *C. fumiferana* in the central and eastern regions. Field studies to examine interspecies interactions with *C. fumiferana* have mostly focused on quantifying introgression with *C. o. biennis* and/or *C. o. occidentalis* within the central region and have discovered limited gene flow (Blackburn et al., 2017; Brunet et al., 2017; Lumley & Sperling, 2011a; Sperling & Hickey, 1995). A northern form of *C. orae* is sympatric with *C. fumiferana* in Alaska and the Yukon (Lumley & Sperling, 2011b; Sperling & Hickey, 1994), and further study of interspecies dynamics in this region would help clarify the likelihood of introgression and its influence on the Western subpopulation.

Only a few studies have identified differences in traits, such as egg weight and number of eggs laid per female, within wild populations of *C. fumiferana* across its geographic range (Harvey, 1983a, 1983b; 1985). Harvey's data (1983a, table I) show striking differences between egg weight for specimens collected in the Central and Eastern regions, where mean initial egg weights ranged from 0.182 to 0.212 mg (mean = 0.196) for locations in the central region (Alberta to Manitoba) and from 0.157 to 0.168 mg (mean = 0.163) for locations in the eastern region (Ontario to New Brunswick), providing an average difference of 0.034 mg. Harvey (1983b) reported in a separate study that insects from Dawson Bay, MB, averaged 0.182 ± 0.002 mg whereas insects reared in the same season collected near Atikokan, ON, averaged 0.171 ± 0.002 mg, a highly significant difference. Given that egg weight and egg number appear to be correlated, this could have a direct impact on population size and, as a consequence, propensity for outbreak. Harvey (1985) also demonstrated that heavier eggs resulted in heavier second‐instar larvae with higher overwintering survival in prolonged cold temperatures. Warming climates will decrease the advantage of laying larger eggs. Movement of genes driving egg size and number from the Eastern subpopulation to the Central subpopulation, combined with warming temperatures and increased survival rates of smaller eggs, may be predicted to lead to more severe outbreaks in the central region. Additional traits that have been reported to vary either across or within geographic regions include morphology (Lorimer, 1982), developmental rates (Shepherd, 1961; Stehr, 1964), and second diapause (Harvey, 1961). We note here that Weber, Volney, and Spence (1999) found no developmental differences in spruce budworm across a latitudinal transect between Cypress Hills, Alberta (49.65 N, −110.03 W) and Old Fort Point, Northwest Territories (64.67 N, −124.90 W). Since their transect was on the eastern side of the mountain ranges that separate the Western and Central subpopulations, the possibility exists that their samples were all from the Central subpopulation and therefore similar in genetics and biology. Additional work to compare northern populations on either side of the mountain range (i.e., Western subpopulation with collections from North West Territories) and to study genotype–environment interactions could help clarify the findings of Weber et al. (1999). In conclusion, our current understanding of if and how *Choristoneura fumiferana* differs in heritable traits across its geographic range is limited, and these differences (e.g., egg weight and size) may have management implications. The delimitation of subpopulations, along with their respective barriers to gene flow, will hopefully provide a roadmap for further examination of their life histories, differing heritable traits, and gene flow.

The present work provides a new understanding of the population genetic structure of *Choristoneura fumiferana* across its geographic range. Although our study has uncovered three subpopulations, little is known about how these subpopulations differ in natural history, including heritable traits. Overall, we recommend that future work should examine differences in heritable traits between the three subpopulations as well as the adaptive consequences of gene flow between them.

## CONFLICT OF INTEREST

None declared.

## AUTHOR CONTRIBUTIONS

LML, MC, and FAHS conceptualized and designed the research. MC, FAHS, and RCL secured funding. LML, EP, and BMTB conducted field sampling and larval rearing. LML, BMTB, and BB developed molecular protocols. LML, EP, and BB conducted laboratory work. LML and JL conducted data analyses. LML wrote the manuscript, with contributions or reviews from all authors. All authors approved the final manuscript.

## Data Availability

Files containing the Fast‐GBS output of biallelic variants, the filtered SNPs data set, and the filtered SNPs with flanking sequences used for analyses in this manuscript are available at the Dryad Digital Repository: https://doi.org/10.5061/dryad.00000000k (Lumley et al., 2019).

## ENDNOTE

1 We have used this naming convention to be consistent with the names established for subpopulations of *Picea glauca* (de Lafontaine et al., 2010), which share a similar geographic distribution. The “Western” subpopulation of *C. fumiferana* should not be confused with the “Western spruce budworm,” which is a different species (*C. occidentalis occidentalis*).
