# Supplementary material for: Continent‐wide population genomic structure and phylogeography of North America’s most destructive conifer defoliator, the spruce budworm (Choristoneura fumiferana)
Source: Ecol Evol. 2020 Jan 7;10(2):914–27. doi: 10.1002/ece3.5950 (PMC6988549; doi:10.1002/ece3.5950)
Supplement: Supplementary file 1 [file ECE3-10-914-s001.pdf]

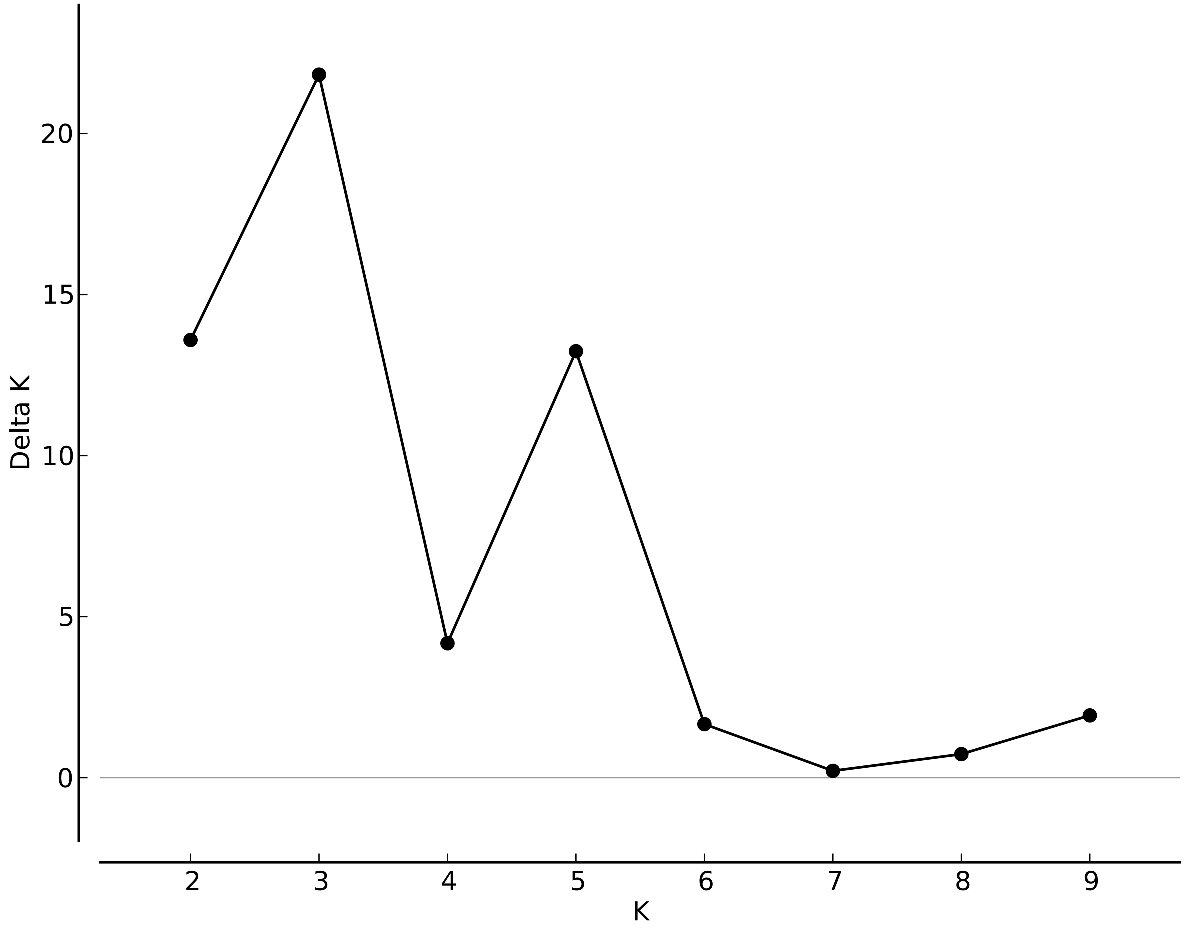

FIGURE S1 Delta K plot for *structure* analysis (K=2 to K=10) of 1975 spruce budworm individuals genotyped for 3650 SNPs.
