# Supplementary material for: Continent‐wide population genomic structure and phylogeography of North America’s most destructive conifer defoliator, the spruce budworm (Choristoneura fumiferana)
Source: Ecol Evol. 2020 Jan 7;10(2):914–27. doi: 10.1002/ece3.5950 (PMC6988549; doi:10.1002/ece3.5950)
Supplement: Supplementary file 2 [file ECE3-10-914-s002.pdf]

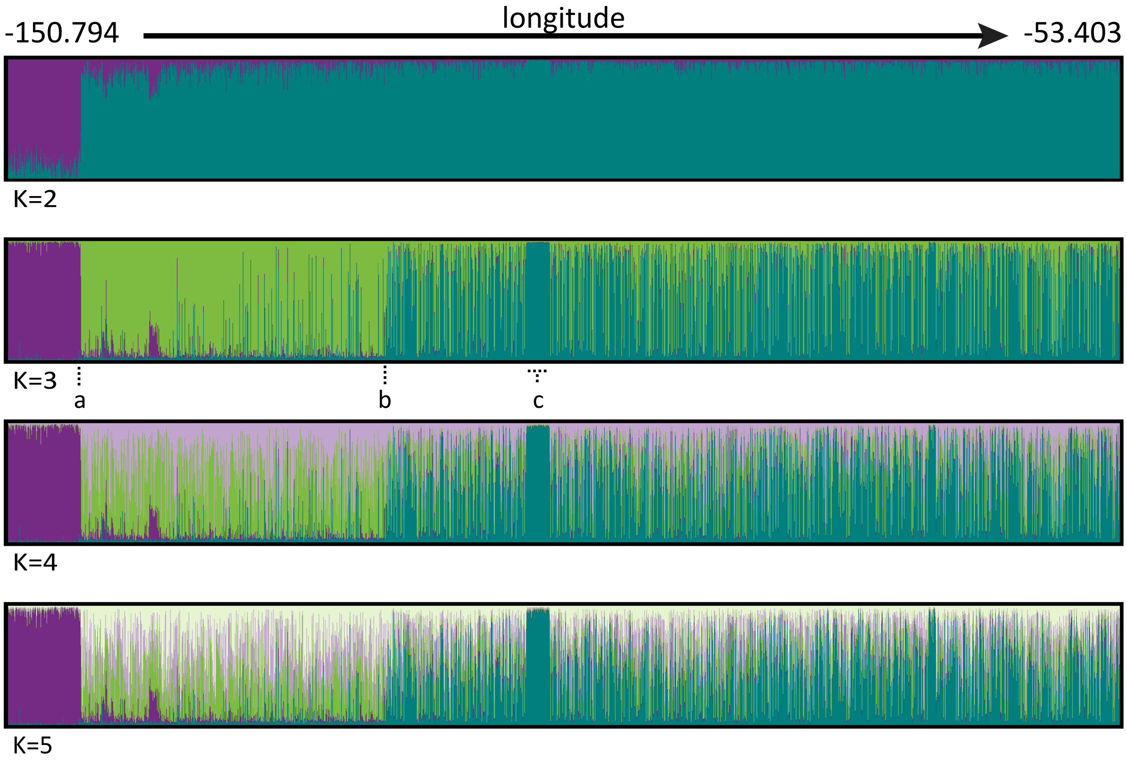

FIGURE S2 Bar plots from *structure* analysis (K=2 to K=5) representing probability of SNP assignment for 1975 spruce budworm individuals analyzed for 3650 SNPs. Individuals are sorted by longitude, with far left representing most western samples (Fish Creek, Alaska) to far right representing most eastern samples (Seal Cove Pond, Newfoundland). Letters along the bottom of the K=3 plot represent the potential geographic barriers to gene flow: a. between Watson Lake and Ross River, Yukon; b. along the Manitoba-Ontario border; and c. the dotted line delimiting all individuals sampled from West Virginia.
