# Supplementary material for: Continent‐wide population genomic structure and phylogeography of North America’s most destructive conifer defoliator, the spruce budworm (Choristoneura fumiferana)
Source: Ecol Evol. 2020 Jan 7;10(2):914–27. doi: 10.1002/ece3.5950 (PMC6988549; doi:10.1002/ece3.5950)
Supplement: Supplementary file 3 [file ECE3-10-914-s003.pdf]

(a)

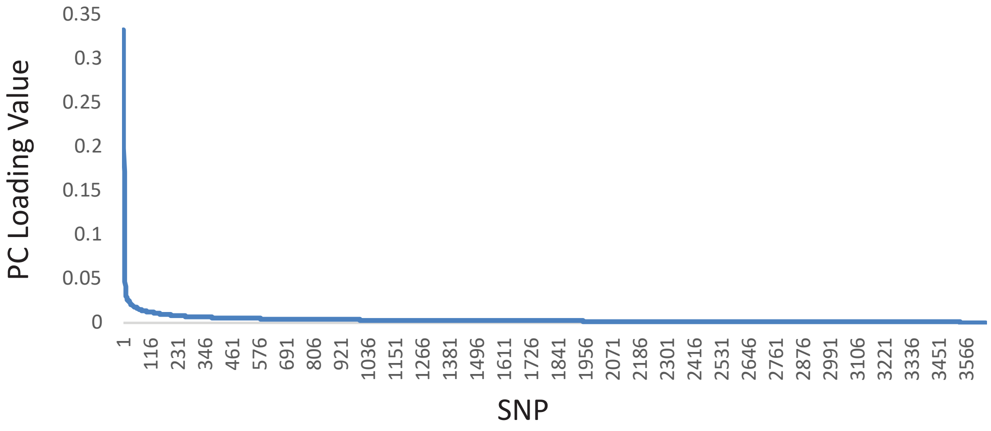

(b)

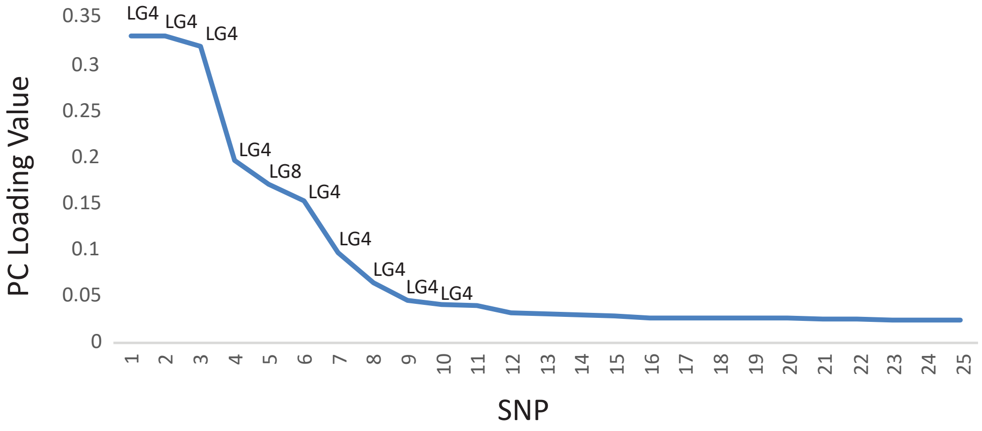

FIGURE S3 PC axis 1 loading values for Central and Eastern spruce budworm subpopulations (1844 individuals, 3650 SNPs) with SNPs ordered from highest to lowest loading value, for: (a) all SNPs, and (b) the 25 highest contributing SNPs, with the first ten labelled with their linkage group.
